# Poseidon’s Trident: “Divine” Intervention in Cervical Cancer Through Chemoradiation, Immunotherapy, and Antibody–Drug Conjugates

**DOI:** 10.3390/cancers18050774

**Published:** 2026-02-28

**Authors:** Yuting Sheng, Hunter E. Wujcik, Mark R. Wakefield, Yujiang Fang

**Affiliations:** 1 Department of Microbiology, Immunology & Pathology, Des Moines University, West Des Moines, IA 50266, USA; yuting.sheng@dmu.edu (Y.S.);; 2 Department of Surgery, University of Missouri School of Medicine, Columbia, MO 65212, USA; wakefieldmr@health.missouri.edu; 3 Ellis Fischel Cancer Center, University of Missouri School of Medicine, Columbia, MO 65212, USA

**Keywords:** immunotherapy, ADCs, cervical cancer 1

## Abstract

**Background/Objectives**: Cervical cancer remains a leading cause of cancer morbidity and mortality worldwide. Although chemoradiation followed by brachytherapy is the curative-intent standard for locally advanced disease, outcomes remain heterogeneous and recurrence and distant metastasis persist. In parallel, immune checkpoint inhibitors (ICIs) and antibody–drug conjugates (ADCs) have expanded systemic options in recurrent or metastatic settings and created new opportunities for multimodality. This review aims to integrate treatment-relevant cervical cancer biology and biomarkers to clarify how chemoradiation, immunotherapy, and ADCs can be optimally selected, sequenced, and combined across disease states. **Methods**: We conducted a structured narrative, evidence-based literature synthesis focusing on cervical cancer management. The review encompassed: (i) the molecular and immune mechanisms underlying human papillomavirus (HPV)-driven carcinogenesis; (ii) contemporary diagnostic and staging approaches, including advanced imaging modalities and histopathological evaluation; and (iii) clinical and translational evidence supporting the optimization of chemoradiation, immune checkpoint inhibition, and antibody–drug conjugates, with emphasis on clinically validated or emerging biomarkers that are relevant to patient stratification and mechanistically rational combination or sequencing strategies. A systematic search of PubMed/MEDLINE, Embase, and major oncology conference proceedings was performed. Priority was given to peer-reviewed original research articles, high-impact clinical trials (Phase II–III), meta-analyses, and consensus guidelines published within the past 10 years to ensure contemporary relevance. Articles published prior to this period were generally excluded to maintain clinical currency; however, seminal studies that established foundational therapeutic standards, mechanistic paradigms, or landmark treatment milestones were intentionally retained due to their enduring influence on current practice. Exclusion criteria included non-peer-reviewed sources, case reports with limited generalizability, non-English publications, and studies lacking methodological rigor or clinical relevance to cervical cancer management. Preclinical studies were included selectively when directly informing therapeutic mechanisms, biomarker development, or translational rationale. This approach was designed to balance historical context with up-to-date clinical applicability, ensuring both scientific rigor and contemporary relevance. **Results**: Chemoradiation and brachytherapy remain essential for local control, while ICIs can restore antitumor T-cell activity in biomarker-enriched contexts. ADCs enable target-directed delivery of potent cytotoxins and may promote immunogenic cell death, supporting immunotherapy and radiation. However, key challenges include resistance mechanisms, toxicity management, and patient identification for the most beneficial combined multimodality. **Conclusions**: A biology- and biomarker-informed framework can guide more rational integration of multimodality therapy in cervical cancer. Future progress will depend on validated predictive biomarkers, optimized sequencing/combination strategies, and trials that balance efficacy with short- and long-term toxicity.

## Simple Summary

Cervical cancer remains a major cause of illness and death worldwide, especially in settings with limited access to prevention, screening, and timely treatment. Standard treatment can cure many patients with localized disease, but some cancers return or spread due to therapeutic resistance or immune system evasion. This review takes a deeper dive into the biological mechanisms of cervical cancer development to examine the rationale behind different treatment strategies—from traditional, standard-of-care chemoradiation therapy to state-of-the-art immunotherapy and targeted drug delivery using antibody–drug conjugates. This review highlights how the interplay between core molecular biology and modern treatment approaches may help to optimize combination strategies, improve outcomes, and ultimately maximize patient care while informing future research directions.

## 1. Introduction

Cervical cancer remains a major global health problem, with disproportionate morbidity and mortality in low- and middle-income countries where access to HPV vaccination, high-quality screening, and timely treatment can be limited [1]. Biologically, most cervical cancers are HPV-driven, and persistent infection with high-risk HPV types establishes a tumor ecosystem shaped by viral oncoproteins (notably E6/E7), chronic inflammation, and progressive immune evasion [2]. This viral etiology is clinically important because it influences tumor antigenicity, host immune contexture, and the biomarker landscape that increasingly guides systemic therapy selection.

For patients with locally advanced disease, the backbone of curative-intent management has traditionally been cisplatin-based concurrent chemoradiation (CCRT), followed by brachytherapy, a sequence designed to maximize local control through radiosensitization and dose escalation to the cervix while respecting normal tissue constraints. Despite refinements in imaging, radiation planning, and supportive care, outcomes remain heterogeneous: some tumors are cured, while others recur locally or develop distant metastases [3]. These patterns highlight persistent unmet needs—particularly for strategies that (i) improve systemic control, (ii) overcome radioresistance or intrinsic chemoresistance, and (iii) address biologic subsets defined by distinct immune and molecular features.

In parallel with advances in radiation delivery and diagnostic imaging, the therapeutic landscape is rapidly evolving with the introduction of immune checkpoint inhibitors (ICIs) and antibody–drug conjugates (ADCs). ICIs have established a role in biomarker-enriched and/or advanced settings by restoring antitumor T-cell activity in tumors that exhibit adaptive immune resistance [4]. ADCs offer a distinct mechanism: target-directed delivery of potent cytotoxins can deepen responses while potentially reshaping the tumor microenvironment through immunogenic cell death and antigen release—creating a rationale for sequence- or combination-based approaches using immunotherapy and radiation [5].

Surgical management remains a cornerstone of cervical cancer treatment, particularly in early-stage disease, where radical hysterectomy and fertility-sparing procedures provide curative intent [6]. While this review synthesizes how molecular and diagnostic information (histopathology, imaging, HPV-associated biology, and clinically used biomarkers) can guide rational use of three major modalities across disease states: chemoradiation, immunotherapy and ADCs. We focus on translating mechanistic insights into practical treatment frameworks—highlighting where evidence is mature, where it is evolving, and which biomarker-driven strategies are most likely to refine patient selection and improve outcomes.

## 2. Materials and Methods

A targeted literature search was performed in PubMed, ClinicalTrials.gov, and the U.S. Food and Drug Administration (FDA) website to identify evidence that is relevant to cervical cancer treatment, including chemoradiation, immunotherapy, and antibody–drug conjugates. The search covered 2010–2025 and prioritized clinically relevant publications (including prospective trials), major regulatory documents, and pivotal studies informing standards of care. Additional relevant references were identified through a manual review of the reference lists. No formal systematic review methodology or meta-analysis was conducted.

## 3. Molecular and Diagnostic Foundations for Treatment

To rapidly operationalize these therapeutic options for consistent clinical benefit, we first need to anchor treatment decisions in the biology and diagnostics that define cervical cancer as a disease: an HPV-driven malignancy with a distinctive immune microenvironment, predictable anatomic routes of spread, and biomarker signals that can inform systemic therapy selection. At the same time, cervical cancer can be devastating to treat once it is invasive and/or metastatic—often requiring multimodality therapy with meaningful acute and long-term toxicities. For that reason, a treatment framework should also begin “upstream”, emphasizing how HPV precancer is identified and manages to prevent progression to malignancy. Accordingly, this section reviews HPV-linked, treatment-relevant tumor biology and immune contexture, the diagnostic tools that define local versus metastatic disease, and the biomarkers that are most likely to impact contemporary treatment selection.

### 3.1. HPV-Driven Biology That Matters for Therapy

Cervical cancers are biologically shaped by persistent infection with high-risk HPV types, where viral oncoproteins—particularly E6/E7—remodel cell-cycle control, DNA damage responses, and antigen presentation pathways. Clinically, the key point is less “how cancer forms” and more how HPV biology changes treatment behavior. In the context of DNA damage, HPV-associated tumors often exist in a distinct DNA damage/repair milieu, which can influence the response to radiation and platinum-based therapy [7]. In terms of antigenicity, viral antigens (e.g., E6/E7-derived peptides) can make tumors more “visible” to the immune system, supporting the rationale for immunotherapy and HPV-targeted immune strategies [8]. However, even with immunologically recognizable antigens, tumors can develop immune evasion mechanisms (e.g., inhibitory checkpoints, impaired antigen presentation), which may help to explain variability in clinical responses to immune checkpoint inhibition [9].

These HPV-linked features help to justify modern approaches that combine local control (radiotherapy (RT) + brachytherapy) with therapies that address systemic risk and immune resistance.

### 3.2. The Tumor–Immune Microenvironment: Hot, Cold, and Excluded Phenotypes

A practical way to translate HPV-linked tumor biology into clinically relevant categories is to describe the cervical tumor–immune microenvironment (TIME) as inflamed (“hot”), non-inflamed (“cold”), or immune-excluded [10]. This framework provides a shared vocabulary for risk stratification and for interpreting variability in immunotherapy responsiveness among tumors with a similar stage and histology.

Inflamed (“hot”) TIME is characterized by intratumoral CD8^+^ T-cell infiltration and interferon/inflammatory signaling, consistent with pre-existing immune engagement. In contrast, non-inflamed (“cold”) TIME is marked by sparse effector T cells and limited evidence of immune priming, whereas immune-excluded TIME features immune cells that are largely restricted to the tumor margin or stroma, with poor penetration into tumor nests [11]. Importantly, the excluded phenotypes are best captured by spatial pathology (cell localization), rather than bulk measures alone.

Clinically, this simplified TIME categorization is most useful for (i) contextualizing biomarker results (e.g., PD-L1 or immune signatures), (ii) informing sampling strategy (spatial assessment when exclusion is suspected), and (iii) guiding interpretation of translational endpoints in CRT–IO or IO-based trials. We return to these concepts in the immunotherapy sections to frame mechanisms of limited benefit and rationale for combination approaches.

### 3.3. Fédération Internationale de Gynécologie et D’Obstétrique (FIGO) Staging and Imaging: Mapping Local Versus Metastatic Disease

While biology increasingly guides systemic therapy, anatomic extent remains the foundation for deciding curative-intent versus palliative-intent strategies. FIGO staging establishes disease extent with emphasis on tumor size, parametrial involvement, vaginal extension, and nodal/distant disease [12]. FIGO staging provides the anatomic framework for disease extent; for local staging and treatment planning, pelvic magnetic resonance imaging (MRI) is central, delineating tumor boundaries, stromal invasion, parametrial involvement, and relationships to bladder/rectum—information that directly informs radiation planning and brachytherapy geometry. Fluorodeoxyglucose positron emission tomography/computed tomography (FDG-PET/CT) further strengthens detection of nodal disease and distant metastases, clarifying whether management should focus on definitive local therapy (CCRT + brachytherapy) with nodal coverage versus systemic therapy-forward strategies.

In practice, imaging and staging determine the therapeutic approach, after which molecular and immune features can refine systemic treatment components [6]. Disease confined to the cervix is typically managed surgically, with definitive radiotherapy reserved for select patients, based on anatomy, comorbidity, or fertility considerations. In contrast, locally advanced tumors—particularly those with parametrial involvement or bulky primary disease (e.g., ≥FIGO IIB)—are generally treated with definitive cisplatin-based chemoradiation, followed by brachytherapy. The identification of pelvic or para-aortic nodal metastases further shapes management by guiding nodal field coverage and escalation, while distant metastatic disease (FIGO IVB) often shifts the overall strategy toward systemic therapy-forward approaches, with radiation used selectively for consolidation or symptom-directed control.

### 3.4. Biomarkers That Matter for Treatment Selection

In cervical cancer, the most clinically actionable biomarkers are those that inform decisions that change management—helping clinicians to select or prioritize systemic therapy, estimate likelihood of benefit, or anticipate resistance. One major category includes immune checkpoint-relevant biomarkers, which capture tumor/immune signaling and immune engagement (for example, PD-L1-based scoring and patterns of immune cell infiltration) and can enrich for response to immune checkpoint inhibitors [13]. A second category reflects genomic instability and DNA repair phenotypes, such as mismatch repair deficiency or high tumor mutational burden (when present), which may support immunotherapy sensitivity in a subset of patients [14,15]. A third group encompasses the target expression for ADCs and other targeted therapies, where expression of specific cell-surface antigens enables antibody-based delivery of potent payloads; importantly, not only the presence of the target but also its level and intratumoral heterogeneity can influence the depth and durability of the response [16]. Finally, pragmatic clinical surrogates—such as baseline tumor burden, nodal status, and early on-treatment response (radiographic or circulating markers)—often correlate with relapse risk and are frequently used to motivate escalation or de-escalation concepts in trials [17]. Across these categories (Table 1), the biomarker value is highest when it directly determines what happens next—choice of therapy, sequencing, or intensity of surveillance—rather than serving as descriptive biology alone.

### 3.5. Treating HPV Precancer: Preventing Malignancy Before It Starts

Because morbidity from invasive cervical cancer treatment can be substantial, prevention and early intervention remain essential components of a modern cervical cancer framework. Primary prevention through HPV vaccination reduces high-risk infections that drive precancer and cancer development [18]. Secondary prevention via screening and triage (high-risk HPV testing, cytology, and risk-based management pathways) identifies individuals who are at elevated risk and directs timely colposcopy and biopsy. High-grade lesions (CIN2/3 and selected CIN2) are managed with either excisional treatment—preferred when invasion must be excluded because it provides tissue for pathology—or ablative approaches in carefully selected settings where invasion is unlikely and follow-up is reliable [19].

Alternatively, fertility and obstetric implications should be incorporated into management. While fertility is generally preserved after treatment of cervical precancer, pregnancy outcomes—particularly preterm birth risk—may be adversely affected, and risk increases with greater cervical tissue removal. A systematic review/meta-analysis (Kyrgiou et al.) reported that women with CIN already have an elevated baseline prematurity risk and that both excisional and ablative treatments are associated with additional risk, with excisional approaches generally conferring higher risk and demonstrating a dose–response relationship with excision depth/volume and repeat procedures [20,21]. Clinically, these data support a fertility-conscious strategy: when oncologically appropriate, ablation may minimize cervical tissue loss; when excision is required, limiting excision depth/volume to what is necessary for adequate margins and avoiding repeat excisions when possible may reduce subsequent obstetric morbidity. Finally, non-procedural approaches (e.g., therapeutic vaccination or other immunomodulatory strategies) for high-grade lesions remain investigational and are being evaluated as potential means to reduce procedural morbidity while maintaining oncologic safety [22].

Taken together, HPV vaccination, screening, and treatment of precancer are not only upstream public-health interventions but also determinants of downstream therapeutic need: by reducing persistent infection and high-grade lesions, they decrease the pool of patients who ultimately present with locally advanced or metastatic disease. These same immunologic principles—antigen recognition, T-cell priming, and immune memory—also provide the conceptual foundation for therapeutic vaccination strategies targeting HPV oncoproteins (E6/E7) in established cancer, bridging prevention biology to therapeutic approaches discussed in the subsequent sections.

## 4. Chemoradiation Backbone

### 4.1. Why Chemoradiation Works as the Mature First-Line Curative Platform

Definitive chemoradiation is the most mature curative-intent backbone for locally advanced cervical cancer because it reliably integrates (i) high-probability local–regional tumor control through radiation dose delivery and (ii) systemic radiosensitization through concurrent chemotherapy [23]. Ionizing radiation produces lethal DNA damage—particularly double-strand breaks—leading to clonogenic cell death via mitotic catastrophe and apoptosis/senescence, depending on context. Concurrent cisplatin enhances this effect by augmenting DNA damage and disrupting repair/tolerance pathways, improving tumor control while maintaining a clinically acceptable therapeutic ratio in appropriately selected patients. In practice, the success of chemoradiation (CRT) hinges less on the concept itself and more on execution: target coverage, nodal management, overall treatment time, and high-quality brachytherapy.

### 4.2. Current Standard: Cisplatin-Based CCRT Plus Image-Guided (Often MRI-Guided) Brachytherapy

Contemporary definitive management typically consists of cisplatin-based concurrent chemoradiation with external beam radiotherapy (EBRT), followed by a brachytherapy boost [24]. The central role of brachytherapy is that it enables steep, conformal dose escalation to the cervix and parametrial tissues while limiting exposure to adjacent organs at risk (bladder, rectum, sigmoid), a dosimetric advantage that EBRT alone generally cannot match safely. Increasingly, image-guided adaptive brachytherapy—often MRI-based—improves target delineation, accounts for tumor regression during treatment, and supports individualized dose sculpting. Practically, this “CCRT + brachytherapy” sequence remains the benchmark regimen that defines modern curative-intent care.

### 4.3. Why This Backbone Remains Essential, Even in the IO/ADC Era

Even as immune checkpoint inhibitors and ADCs expand systemic options, chemoradiation remains indispensable for three reasons. First, durable survival for many patients still requires definitive pelvic control, and failure to control the primary tumor and regional disease can be immediately life-limiting. Second, anatomic extent at presentation (tumor bulk, parametrial involvement, nodal disease) is common, making a purely surgical approach infeasible for many patients; CRT provides a standardized, scalable strategy across these presentations. Third, the newest systemic advances have largely been validated as add-ons, rather than replacements for definitive CRT: for example, the pivotal KEYNOTE-A18/ENGOT-cx11 phase III trial evaluated pembrolizumab added to chemoradiotherapy in high-risk locally advanced disease—reinforcing CRT as the foundational platform, even as outcomes improve with immunotherapy integration [25]. Similarly, ADCs have established activity primarily in the previously treated recurrent/metastatic setting, rather than as curative substitutes; in the pivotal innovaTV-204 study of tisotumab vedotin monotherapy after progression in chemotherapy, the confirmed objective response rate was ~24% (95% CI ~16–33%), with a median duration of response of ~8.3 months, highlighting meaningful but non-curative activity in a refractory population [26]. Finally, high-quality brachytherapy remains a unique, non-substitutable component of cure within definitive radiotherapy programs, and major guidelines explicitly caution against replacing image-guided brachytherapy with an external-beam boost.

### 4.4. Radiosensitizers and Future Chemo–RT Refinements

Cisplatin remains the prototypical concurrent radiosensitizer during definitive chemoradiation, but ongoing refinements aim to improve the therapeutic ratio and mitigate distant relapse risk. These efforts include optimization of cisplatin scheduling and supportive care to maintain dose intensity and treatment compliance, with multiple analyses comparing weekly versus triweekly cisplatin regimens and their toxicity/completion profiles [27]. In parallel, alternative concurrent approaches for cisplatin-ineligible patients (e.g., weekly carboplatin) are incorporated into contemporary practice guidance, while risk-adapted strategies seek to intensify systemic therapy around CRT for high-risk features (such as bulky tumors or nodal disease), informed by large randomized trials evaluating additional chemotherapy either after CRT (OUTBACK) or immediately before CRT (INTERLACE).

### 4.5. Bridge to the Immunotherapy Section: Why CRT Is a Logical Partner for IO

**Figure 1:**
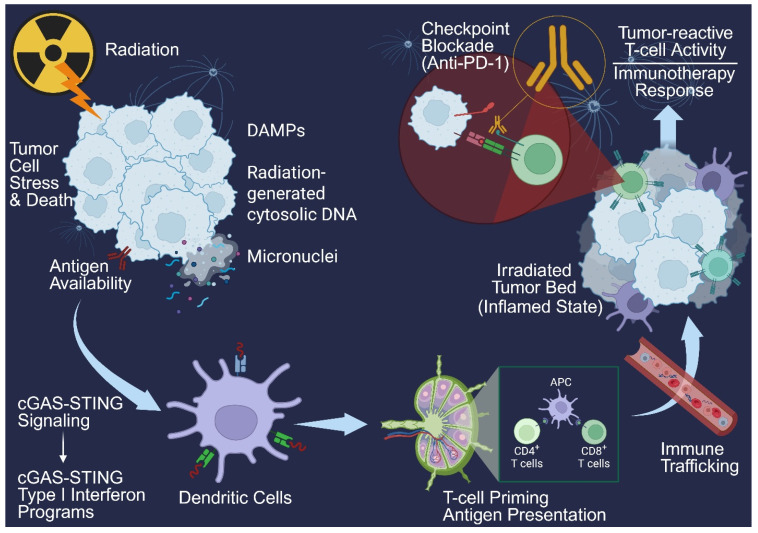
Radiotherapy-induced immune activation and synergy with anti-PD-1. Schematic overview of how radiotherapy can promote antitumor immune responses through immunogenic tumor cell stress and death. Radiation increases antigen availability and releases damage-associated molecular patterns (DAMPs) while generating cytosolic DNA and micronuclei, which activate cGAS–STING signaling and downstream type I interferon programs. These signals support dendritic cell activation and antigen presentation, leading to CD4^+^ and CD8^+^ T-cell priming, immune trafficking into an inflamed irradiated tumor bed, and enhanced tumor-reactive T-cell activity. The anti-PD-1 checkpoint blockade is depicted as augmenting T-cell effector function, contributing to improved immunotherapy response. Created in BioRender. Sheng, Y. (2026) https://BioRender.com/7k0y3z3.

Chemoradiation is also an important conceptual lead-in to immunotherapy because radiation can do more than cytoreduction. RT-induced tumor cell stress and death can increase antigen availability and release damage-associated molecular patterns (DAMPs), promoting dendritic-cell activation and T-cell priming—features often described as immunogenic cell death [28]. In addition, radiation-generated cytosolic DNA and micronuclei can activate cGAS–STING signaling and downstream type I interferon programs, which may enhance antigen presentation and immune trafficking in the irradiated tumor bed. Together, these effects provide a biologically plausible rationale for the hypothesis that CRT may “condition” the tumor microenvironment in ways that are potentially relevant to checkpoint blockade and other immune-based strategies, although the extent of clinical translation remains under active investigation, motivating the IO-focused discussion that follows, illustrated in Figure 1. This suggests that CRT may function as an “immune primer”—helping to convert immunologically cold or excluded tumors toward a more inflamed state, thereby creating a substrate for checkpoint blockade to amplify and sustain tumor-reactive T-cell activity.

In parallel, another hypothesis that resides in the bridge to ADCs is conceptually distinct: rather than primarily “unbraking” immunity, ADCs provide target-directed cytotoxic intensification that may enhance tumor control and address micro-metastatic disease while remaining compatible with definitive local therapy, with any immune stimulation from payload-mediated immunogenic death serving as a complementary effect [29].

Importantly, framing CRT as the current backbone should not be interpreted as a barrier to innovation. Rather, as shown in Table 2, CRT provides a clinically proven platform upon which newer modalities—immunotherapy and ADCs—can be layered, optimized, and validated as evidence matures. The field’s goal is not to pit CRT against IO or ADCs as competing “winners”, but to integrate these modalities rationally so that each contributes its strongest advantage: CRT for definitive local control, and systemic agents for micro-metastatic disease control and biologically informed personalization. In this sense, modern cervical cancer therapy is less a single replacement pathway and more a coordinated “trident”—three complementary prongs working in concert toward the shared objective of improving cure rates, durability of response, and quality of survivorship.

## 5. Immunotherapy

### 5.1. The Perfect Setup

Despite its efficacy as the cornerstone of local intervention, chemoradiation does not inherently induce systemic immunity. This limitation has driven the investigation of complementary strategies that are capable of generating durable, systemic antitumor responses [30]. Immunotherapy (IO), by leveraging the foreign viral antigens inherent to HPV-positive tumors, represents a paradigm-shifting approach that directly addresses this therapeutic gap [31].

Remarkably, the immunogenicity of human papillomavirus-positive (HPV+) cervical cancer creates a favorable therapeutic context for immunotherapy. The constitutive expression of viral oncoproteins (E6/E7) generates a robust, pre-existing population of tumor-specific T-cells. However, this immune response is actively suppressed by the tumor’s upregulation of the Programmed Death-Ligand 1 (PD-L1), which engages the Programmed Death-1 (PD-1) receptor on T-cells, inducing exhaustion [32]. Therapeutically blocking the PD-1/PD-L1 interaction releases this suppression, reinvigorating anti-tumor T-cell activity and unleashing a potent immune-mediated attack [33].

### 5.2. Key Agents and Trials

Currently, among immune checkpoint inhibitors explored in cervical cancer, pembrolizumab, a monoclonal antibody that targets and blocks the PD-1 receptor, has the most mature clinical evidence, being the most extensively studied agent and the only one with published phase III results [34]. In the randomized, double-blind phase III KEYNOTE-826 trial, patients with persistent, recurrent, or metastatic cervical cancer were assigned (1:1) to receive pembrolizumab 200 mg every 3 weeks (up to 35 cycles) or a placebo in combination with platinum–taxane chemotherapy, with optional bevacizumab at the investigator’s discretion [35]. PD-L1 combined positive score (CPS)—a PD-L1 immunohistochemistry metric incorporating staining in both tumor and immune cells—was used for prespecified efficacy analyses. Dual primary endpoints were progression-free survival (PFS) and overall survival (OS), tested sequentially in the PD-L1 CPS ≥ 1 population, the intention-to-treat population, and the PD-L1 CPS ≥ 10 population.

At the protocol-specified first interim analysis, adding pembrolizumab produced clinically meaningful improvements in both PFS and OS. In the PD-L1 CPS ≥ 1 population (*n* = 548), median PFS was 10.4 vs. 8.2 months (hazard ratio (HR) 0.62; 95% CI, 0.50–0.77; *p* < 0.001) and 24-month OS was 53.0% vs. 41.7% (HR 0.64; 95% CI, 0.50–0.81; *p* < 0.001) for pembrolizumab vs. placebo, respectively [35]. A similar benefit was seen in the intention-to-treat population (*n* = 617) (PFS HR 0.65; OS HR 0.67), and in the PD-L1 CPS ≥ 10 subgroup (*n* = 317) (PFS HR 0.58; OS HR 0.61). The most common grade 3–5 adverse events were anemia (30.3% vs. 26.9%) and neutropenia (12.4% vs. 9.7%) [35].

Collectively, phase III data support pembrolizumab in two major settings: (1) persistent/recurrent/metastatic PD-L1 CPS ≥ 1 disease in combination with chemotherapy (±bevacizumab), and (2) FIGO 2014 stage III–IVA locally advanced disease in combination with chemoradiotherapy. Importantly, these approvals retain established curative-intent backbones (chemotherapy and/or chemoradiotherapy), underscoring that single-agent IO in cervical cancer has more limited efficacy and fewer mature outcomes compared with combination strategies.

Interestingly, even where pembrolizumab has demonstrated phase III benefit, it has been incorporated as an adjunct to established curative-intent backbones (chemotherapy and/or chemoradiotherapy), reflecting that IO monotherapy has not yet demonstrated sufficient efficacy to replace these first-line curative approaches; this limitation is illustrated by the earlier monotherapy experience in KEYNOTE-158, a multicenter, non-randomized, open-label, phase II “basket” trial that included a dedicated cohort of patients with recurrent or metastatic cervical cancer, previously treated with systemic therapy. Patients received pembrolizumab 200 mg IV every 3 weeks for up to 2 years or until progression/toxicity; the primary endpoint was the objective response rate (ORR) [36]. Among 98 treated patients, most tumors were PD-L1 positive by CPS ≥ 1. The study reported a modest but notable response signal with durability: the ORR was 12.2% overall (including three complete and nine partial responses), and all responses occurred in PD-L1-positive tumors (ORR 14.6% in PD-L1 CPS ≥ 1). The median duration of response was not reached at the time of reporting, suggesting that a subset of patients can derive durable benefit despite the low overall response rate. However, this activity remains limited and is accompanied by clinically meaningful toxicity: serious adverse reactions occurred in 39% of patients, and treatment discontinuation due to adverse reactions was reported, underscoring the need for careful patient selection and close monitoring. Taken together, these findings suggest that pembrolizumab monotherapy remains a suboptimal strategy relative to combination approaches, with benefit confined to a minority of patients.

In contrast to pembrolizumab—where monotherapy evidence in cervical cancer is largely derived from single-arm phase II data with modest response rates (KEYNOTE-158)—cemiplimab (Libtayo) provides one of the strongest examples of IO monotherapy supported by randomized phase III survival benefit in the later-line setting. In EMPOWER-Cervical 1/GOG-3016/ENGOT-cx9, 608 women with recurrent cervical cancer after first-line platinum therapy were randomized (1:1) to cemiplimab 350 mg IV every 3 weeks or investigator’s-choice single-agent chemotherapy [37]. Cemiplimab significantly improved overall survival (median 12.0 vs. 8.5 months; HR 0.69, 95% CI 0.56–0.84; *p* < 0.001) and improved the objective response rate (16.4% vs. 6.3%) compared with chemotherapy [38]. Notably, responses were observed in both PD-L1 ≥ 1% and PD-L1 < 1% subgroups, supporting activity that is less dependent on PD-L1 enrichment than pembrolizumab monotherapy signals. In Europe, cemiplimab is indicated as monotherapy for recurrent/metastatic cervical cancer progressing on/after platinum therapy; however, the cervical cancer application was voluntarily withdrawn in the U.S., underscoring how regional regulatory context can shape clinical adoption [39].

Despite positive phase III survival data supporting cemiplimab monotherapy in the post-platinum recurrent/metastatic setting, its U.S. regulatory pathway diverged from Europe. In January 2022, the sponsors voluntarily withdrew the U.S. supplemental BLA for cemiplimab in advanced cervical cancer after reporting that they were unable to align with the FDA on required post-marketing studies, and the cervical cancer indication therefore remains not FDA-approved in the United States. The sponsors also noted the evolving U.S. treatment landscape following approval of another PD-1 inhibitor in the first-line setting, which may narrow the practical niche for second-line PD-1 monotherapy. In this context, it is important to distinguish the evidentiary basis for monotherapy strategies: pembrolizumab is established in the first-line setting as combination therapy, while single-agent activity has been supported primarily by single-arm phase II data, which demonstrated only limited efficacy to a specific patient population and clinically meaningful toxicity, as observed. By contrast, cemiplimab monotherapy demonstrated an overall survival advantage versus investigator’s-choice chemotherapy in a randomized phase III trial.

Beyond these regulatory and evidentiary differences, continued interest in IO monotherapy stems from its potential to provide a reduced cytotoxic systemic approach that may decrease select long-term treatment-related toxicities that are relevant to survivorship, including reproductive health. Because immune checkpoint inhibitors are not conventional cytotoxic agents, immunotherapy has been hypothesized to avoid certain long-term toxicities associated with cytotoxic therapy (including infertility), although direct oncofertility data for ICIs remain limited and are still emerging [40]. Accordingly, immunotherapy monotherapy has been proposed as a potentially fertility-sparing systemic option for select patients of childbearing age compared with cytotoxic therapy, pending validation in studies with robust reproductive endpoints. Yet, fertility preservation counseling remains important, as reproductive outcome data in this setting are limited and prospective registries are needed. Therefore, this consideration is clinically relevant because the 2019 Global Burden of Disease study reported that 45.4% of cervical cancer cases occurred among women aged 15–49 years (256,900 of 565,541 cases) [41].

### 5.3. Biomarkers and Resistance Mechanisms

Recent advances in immuno-oncology for cervical cancer are encouraging; however, the clinical benefit of immune checkpoint blockade remains constrained by biologic specificity—namely, whether a patient’s tumor harbors the antigenic and microenvironmental features required for effective immune restoration. In practice, this translates into patient selection, for which biomarkers provide the principal framework. Among the biomarker domains that are most relevant to cervical cancer are HPV-related immune biology, PD-L1 expression quantified by the CPS, and broader TIME phenotypes.

Because approximately 95% of cervical cancers arise in the setting of HPV infection, IO biomarker development in this disease naturally extends beyond PD-L1 to include viral antigen-driven immune features [42]. In HPV-associated tumors, evidence of HPV antigenicity—particularly expression of the viral oncoproteins E6 and E7 and the presence of E6/E7-specific T-cell responses—is frequently invoked as a mechanistic rationale for immunotherapeutic vulnerability, given that these viral genes are central drivers of malignant transformation [43]. In addition to E6/E7, CD8-positive (CD8^+^) T-cell infiltration is another HPV-linked biomarker that is of particular interest, as increased CD8^+^ T-cell density is commonly interpreted as a “hot” phenotype that is more permissive to checkpoint blockade through pre-existing antitumor immunity [44]. Consistent with this concept, Zhu et al. identified a PD-1+ and 4-1BB-positive (4-1BB+) CD8^+^ tumor-infiltrating lymphocyte (TIL) subset enriched within cervical tumors (median 2.59% of CD8^+^ TILs vs. 0.14% in peripheral blood; *p* < 0.001) [45]. Consistently, a PD-1/4-1BB “high” transcriptional signature stratified patients with improved disease-specific survival (DSS) and progression-free interval (PFI) in The Cancer Genome Atlas (TCGA) cohort (DSS *p* = 0.03; PFI *p* = 0.02) and was associated with higher predicted benefit from immune checkpoint blockade. The PD-1/4-1BB high subgroup also exhibited a more inflamed immune contexture, including increased expression of cytotoxic effector genes (e.g., interferon gamma [IFNG], granzyme B [GZMB], and perforin 1 [PRF1]), supporting the utility of this composite CD8^+^ “tumor-reactive/activatable” phenotype as a biologically grounded biomarker concept for IO responsiveness.

Building on HPV-driven antigenicity and T-cell infiltration, the most widely implemented clinical biomarker in cervical cancer immunotherapy is PD-L1; its clinical relevance in immuno-oncology is clearest in how it is operationalized for checkpoint blockade patient selection [46,47]. In current U.S. practice, PD-L1 expression quantified by the combined positive score (CPS)—which incorporates PD-L1 staining on both tumor cells and tumor-associated immune cells normalized to the total number of viable tumor cells—is the principal pretreatment biomarker used to guide pembrolizumab use in cervical cancer-specific indications. Specifically, pembrolizumab is indicated (i) in combination with chemotherapy with or without bevacizumab for persistent, recurrent, or metastatic disease and (ii) as monotherapy after progression on chemotherapy only for PD-L1-positive tumors (CPS ≥ 1) as determined by an FDA-approved assay, most commonly PD-L1 immunohistochemistry (IHC) 22C3 pharmDx as the companion diagnostic [48,49].

Finally, tumor immune microenvironment (TIME) biomarkers encompass many of the factors discussed above because TIME is an umbrella concept that evaluates the tumor as an immune ecosystem, rather than a single marker. Importantly, TIME profiling also extends beyond response prediction to address mechanisms of resistance to immunotherapy (IO) by identifying microenvironmental features associated with immune evasion in specific patient subsets. Accordingly, this section focuses on TIME-based patterns that may predict suboptimal IO benefit and highlights clinical contexts in which patients may warrant alternative or combination treatment modalities. First and foremost, a common category of TIME often used to evaluate T-cell exhaustion/dysfunction is the combination of PD-1, T-cell immunoglobulin and mucin-domain containing protein 3 (TIM-3; *HAVCR2*), Lymphocyte activation gene 3 (LAG-3; *CD223*), and T-cell immunoreceptor with immunoglobulin and ITIM domains (TIGIT) [50]. PD-1 is the most commonly used exhaustion-associated checkpoint; however, it alone is not specific because it can also mark recent activation. Therefore, TIM-3 is frequently evaluated with PD-1 because it is enriched on more dysfunctional/terminally exhausted tumor-infiltrating T cells, and PD-1+TIM-3+ co-expression is widely used as a pragmatic marker of deeper exhaustion than PD-1 positivity alone; in a study done by Jin et al., it demonstrated “cooperation” of TIM-3 and PD-1 in CD8 T-cell exhaustion during chronic viral infection and uses PD-1+TIM-3+ as an exhaustion-associated subset [51]. In addition, LAG-3 is widely incorporated into multiparameter exhaustion panels because LAG-3 is enriched on dysfunctional tumor-infiltrating T cells, frequently co-expressed with PD-1, and has been shown to help distinguish functionally exhausted PD-1+LAG-3+ subsets from more functional PD-1+LAG-3− T cells [52]. Furthermore, TIGIT is often included in T-cell exhaustion/dysfunction panels because it is upregulated on a chronically stimulated tumor-infiltrating cluster of differentiation 8–positive (CD8^+^) T cells, and by competing with the activating receptor CD226 (*DNAM-1*) for ligands such as CD155 (poliovirus receptor, *PVR*), it transmits dominant inhibitory signals associated with reduced proliferation/cytokine production and impaired anti-tumor effector function [53]. In a cervical cancer-specific study, García-Barrientos et al. used multiparametric flow cytometry and immunohistochemistry to show that tumor-infiltrating clusters of differentiation 8–positive (CD8^+^) T cells frequently co-express multiple inhibitory receptors—most prominently, PD-1 and TIGIT (median ~54% PD-1+TIGIT+ in tumor-infiltrating lymphocytes), with additional enrichment of PD-1+TIM-3 and PD-1+LAG-3 populations—supporting a highly immunosuppressive, exhaustion-like TIME [54]. Notably, locally advanced tumors exhibited higher TIGIT and lower CD8^+^ infiltration, which is consistent with a microenvironment that may be less amenable to PD-1/PD-L1 blockade alone; accordingly, such “multi-checkpoint-high” phenotypes may be most appropriate for combination strategies (e.g., dual-checkpoint approaches or IO plus standard modalities), rather than reliance on single-agent checkpoint inhibition pending prospective validation. Notably, although multi-parameter exhaustion markers (e.g., TIM-3, LAG-3, TIGIT) are increasingly studied, they are not currently incorporated into major cervical cancer clinical guidelines as routine decision-making biomarkers and are therefore primarily used in translational analyses and clinical trial stratification.

To enhance translational clarity, Table 3 summarizes how the FIGO stage establishes the treatment backbone and how PD-L1 status, TIME phenotype, HPV-related immune features, and ADC target expression may inform modality selection and sequencing, with investigational elements explicitly labeled.

### 5.4. Next Wave

Beyond checkpoint blockade, two emerging IO directions in cervical cancer include HPV-directed therapeutic vaccination and adoptive TIL therapy. Therapeutic vaccines (often targeting HPV16 E6/E7) are designed to expand HPV-specific T-cell immunity and may synergize with immune checkpoint inhibitors by increasing the pool of tumor-reactive T cells available for checkpoint-mediated functional restoration; early clinical studies of vaccine–checkpoint combinations (e.g., ISA101-based regimens and VB10.16 plus atezolizumab) have reported encouraging activity and durable responses in HPV16-positive advanced disease [55,56]. In parallel, TIL therapy (e.g., LN-145) offers a cell-based approach that can generate meaningful responses in heavily pretreated cervical cancer, highlighting adoptive cellular therapy as a potential option for patients with refractory disease, albeit with important manufacturing and toxicity considerations [57,58].

## 6. Antibody–Drug Conjugates

### 6.1. The Best of Both Worlds

By integrating the strengths of the two therapeutic paradigms discussed above, ADCs offer a strategy that couples tumor-selective immune-guided targeting with delivery of a potent cytotoxic payload, thereby preserving the efficacy of chemotherapy while improving precision. In this way, ADCs function as a “targeted chemotherapy” platform that can also modulate the tumor immune microenvironment through immunogenic cell death and enhanced antigen release, providing a biologic rationale for combination with immunotherapy.

Consequently, ADCs have emerged as one of the most active and rapidly evolving areas in contemporary cervical cancer treatment. ADCs function as a targeted delivery platform that couples a tumor-selective antibody to a highly potent cytotoxic payload. This “guided” approach enables the clinical use of agents that would otherwise be prohibitively toxic if administered systemically, by concentrating drug delivery within antigen-expressing tumor tissue and thereby limiting off-target exposure. In addition, the payload is attached via a chemical linker engineered to remain stable in the circulation and to minimize premature drug release, improving the pharmacokinetic stability and reducing unintended systemic toxicity [59].

### 6.2. New Kid on the Block

Currently, the most promising agent among ADC is tisotumab vedotin, a tissue factor (TF)-directed agent composed of a fully human immunoglobulin G1 kappa (IgG1κ) anti-TF monoclonal antibody linked to the microtubule-disrupting payload monomethyl auristatin E (MMAE) via a protease-cleavable maleimidocaproyl–valine–citrulline–p-aminobenzyl carbamate (mc–Val–Cit–PABC) linker; after TF binding, the conjugate is internalized and lysosomal processing releases MMAE, enabling targeted intracellular cytotoxicity [60].

Contributing to its distinctive therapeutic profile, tisotumab vedotin (TV) couples tumor-directed targeting with delivery of a highly potent cytotoxic payload, thereby enhancing chemotherapy-like efficacy while aiming to reduce nonspecific systemic exposure. This clinical benefit is supported by the pivotal innovaTV 301 trial, in which 502 patients with recurrent or metastatic cervical cancer experienced significantly improved overall survival with TV compared with investigator’s choice chemotherapy (median 11.5 vs. 9.5 months), corresponding to a 30% relative reduction in the risk of death (hazard ratio 0.70, 95% confidence interval 0.54–0.89; two-sided *p* = 0.004) [61]. Nevertheless, despite its efficacy, TV has thus far been positioned primarily as a second- or third-line therapy, in part because treatment-limiting toxicity remains clinically meaningful: 14.8% of patients discontinued TV due to adverse events, including ocular toxicity, peripheral neuropathy, and bleeding. Among these, ocular and mucosal toxicities are particularly distinctive and clinically challenging, and are thought to reflect an on-target, off-tumor effect—consistent with TF expression on certain non-malignant ocular surfaces and mucosal tissues—highlighting the importance of proactive mitigation strategies and next-generation designs aimed at improving tumor selectivity [62,63]. However, supportive care protocols and prophylactic measures are now well established—and continue to evolve—to accompany ADC therapy and mitigate predictable toxicities.

### 6.3. Why Is ADC Still Not First-Line

Antibody–drug conjugates (ADCs) represent an important class of targeted cancer therapies, but substantial challenges remain [64]. These include tumor antigen heterogeneity, resistance mechanisms, systemic toxicities, and difficulties in clinical translation that may limit efficacy.

Further defining the challenges described in the last paragraph, moving ADCs earlier in the treatment course raises important safety and implementation concerns. ADC-related toxicities may overlap with or compound chemoradiation- or immunotherapy-associated adverse events, potentially limiting dose intensity, prolonging treatment, or increasing interruptions in curative-intent regimens [65]. In multimodality settings, careful attention to scheduling (concurrent vs. sequential), organ-at-risk toxicity (e.g., ocular, hematologic, pulmonary, or neuropathic effects, depending on payload/target), and supportive-care feasibility is essential [66,67]. Accordingly, the trial design should therefore incorporate conservative dose-escalation or safety run-in phases, prespecified stopping rules, and robust monitoring of both acute and late effects, including patient-reported outcomes and survivorship endpoints. On the other hand, biomarker considerations are also central: intratumoral target heterogeneity and dynamic antigen loss may reduce the benefit, supporting the need for standardized assays, threshold definitions, and correlative sampling (including on-treatment biopsies and/or ctDNA, where feasible) [68,69]. In sum, these issues highlight that earlier-stage ADC strategies require rigorous prospective evaluation to define patient selection, sequencing, and risk–benefit trade-offs before adoption beyond clinical trials.

Another reason ADCs have not yet moved into the first-line setting is therefore less about the feasibility of management and more about the current evidence base: for tisotumab vedotin (TV) and most ADCs in cervical cancer, efficacy has been demonstrated predominantly in the post-chemotherapy population. To justify first-line use, ADCs generally require phase III, head-to-head data showing improved outcomes versus established first-line regimens with an acceptable safety profile. Nonetheless, multiple early-phase studies (phase Ib/II) are already evaluating TV in combination strategies and earlier lines of therapy, providing a rationale—and momentum—for definitive phase III trials that could ultimately reposition ADCs as first-line options [70].

Further positioning TV as a mainstream therapy in cervical cancer will likely depend on progress along two fronts. First, definitive phase III comparative trials are needed to establish how TV-containing strategies perform against the current standards—both conventional chemoradiotherapy platforms and immunotherapy-based regimens—in clinically relevant populations. Second, given the higher safety and tolerability standards required in earlier-line settings, reducing the clinical impact of TV’s ocular toxicity (and, ultimately, the rationale for its boxed warning) remains an important prerequisite for broader first-line adoption.

Multiple phase Ib/II studies are moving TV earlier in the treatment paradigm, while parallel efforts have refined supportive care algorithms and prophylaxis—particularly for ocular adverse events—thereby improving its feasibility in routine practice. At the same time, the field may benefit from complementing these “passive” mitigation strategies with “active” approaches that enhance tumor selectivity at the level of drug activation. Because TF is not tumor-exclusive and can be expressed on ocular surface and mucosal tissues, next-generation designs could shift the emphasis from target recognition alone to conditional payload release, whereby cytotoxic activation requires tumor-associated protease activity to cleave a protease-sensitive linker and liberate the payload. Under this paradigm, TF-expressing tumor cells would be more likely to support productive activation and intracellular payload release, whereas TF-expressing normal tissues lacking the requisite tumor-associated protease milieu would be less likely to trigger drug liberation, potentially reducing on-target, off-tumor toxicity [71]. Proof-of-concept for this conditional activation strategy comes from first-in-human evaluation of protease-activatable “masked” antibody–drug conjugates. In the PROCLAIM-CX-2029 dose-escalation trial (NCT03543813), CX-2029—a protease-activatable (masked) anti-CD71 Probody–drug conjugate bearing a MMAE payload—was administered every three weeks across 0.1–5 mg/kg in 45 patients with advanced solid tumors [72]. Dose-limiting toxicities emerged at higher doses (including febrile neutropenia/pancytopenia at 5 mg/kg and additional DLTs at 4 mg/kg), leading to the selection of 3 mg/kg as the recommended phase II dose. Among 37 response-evaluable patients, three confirmed partial responses were observed (overall response rate 8.1%)—all in squamous histologies—and durable disease control (partial response or stable disease ≥ 16 weeks) occurred in 12/37 (32.4%). Pharmacokinetic analyses showed that the conjugate circulated predominantly as intact masked ADC (>90%) with low free MMAE (≤4.3%), supporting the feasibility of conditional tumor-site activation to improve selectivity for broadly expressed targets [73].

In addition to the above-described protease-activated “masked antibody” approaches, another strategy being explored to reduce on-target, off tumor toxicity is “AND-gate” dual-antigen targeting with bispecific ADCs. These designs aim to increase specificity by requiring co-expression of two antigens (often via avidity) to achieve strong binding/internalization, rather than targeting cells expressing either antigen alone [74]. However, translating AND-gate bispecific ADCs into clinically deployable therapeutics introduces substantial engineering and regulatory complexity, including challenges in bispecific antibody assembly, conjugation control (e.g., consistent drug-to-antibody ratio and stability), and reproducible manufacturing/chemistry, manufacturing, and control specifications [75]. In addition, regulators typically expect robust evidence that the two-antigen “logic” performs as intended in relevant human tissues, often necessitating validated assays (and potentially companion diagnostics) to quantify co-expression patterns, define thresholds, and anticipate heterogeneity-driven escape. Consequently, despite this compelling theoretical rationale for improving tumor selectivity through dual-antigen (“AND-gate”) targeting, this concept has not yet been implemented for TV, which remains a tissue factor (CD142)-only platform. Accordingly, an additional tumor-enriched antigen partner for TF has not been established in the context of TV and represents an important opportunity for future development.

In the following paragraph, we propose several plausible TF + X pairings grounded in cervical cancer biomarkers biology that could be prioritized in preclinical studies, with the long-term goal of enabling safer and more effective TF-directed strategies that are capable of moving beyond later-line use and toward earlier-line—and potentially first-line—therapy. Cervical cancer profiling studies have highlighted the substantial expression of additional antibody–drug conjugate targets—most notably, trophoblast cell-surface antigen 2 (TROP2) and nectin-4—including reports of TF co-expression with these candidates in primary and metastatic lesions, supporting their consideration as ‘second-antigen’ partners for TF-directed platforms [76,77]. Other candidates such as folate receptor alpha (FRα/*FOLR1*) and B7-H3 may define biologically distinct subsets and could be integrated into dual-target designs to improve tumor selectivity and mitigate normal-tissue binding, pending systematic tumor-versus-ocular expression mapping [78,79].

### 6.4. ADC and IO Synergy

Looking ahead, tisotumab vedotin has the potential to move earlier in the treatment paradigm if ongoing development can preserve its efficacy while further improving tolerability. At present, however, the most actionable path is not ADC monotherapy in the first-line setting, but rational combination strategies, leveraging ADCs as complementary agents that may deepen and prolong responses through rational immune modulation when paired with immunotherapy.

**Figure 2:**
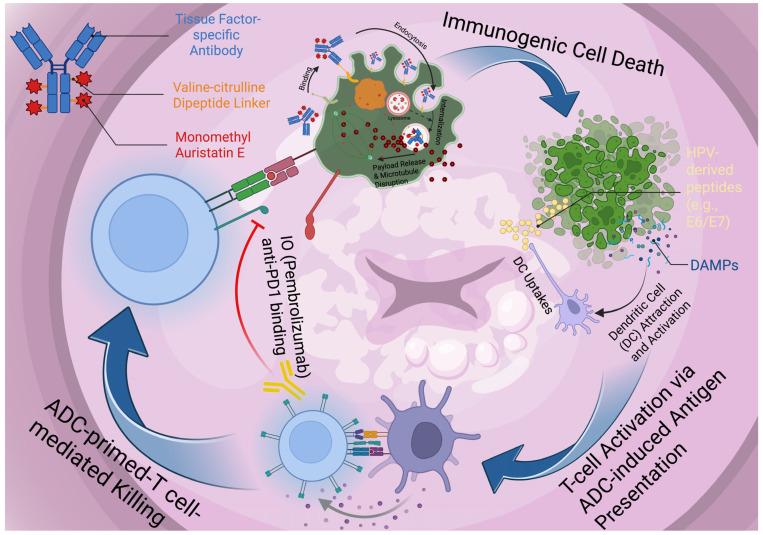
ADC-mediated immune priming and synergy with PD-1 blockade in cervical cancer. A tissue factor (TF)-targeting antibody–drug conjugate (ADC; valine–citrulline linker conjugated to monomethyl auristatin E [MMAE]) binds TF on tumor cells, undergoes endocytosis, and releases payload intracellularly, leading to microtubule disruption and tumor-cell death with immunogenic features. Immunogenic cell death promotes the release of damage-associated molecular patterns (DAMPs) and tumor/HPV-derived antigens (e.g., E6/E7 peptides), which are captured by dendritic cells (DCs), driving DC activation and antigen processing/cross-presentation. These events prime and expand tumor-reactive T cells that mediate ADC-primed T-cell killing. Concurrent PD-1 blockade (pembrolizumab) enhances effector T-cell function by preventing inhibitory PD-1 signaling, reinforcing a feed-forward cycle of antigen release, T-cell activation, and tumor clearance. Created in BioRender. Sheng, Y. (2026) https://BioRender.com/iis0tji. These immunogenic and immunostimulatory properties provide a strong mechanistic rationale for combining ADCs with immune checkpoint blockade, including PD-1/ PD-L1 inhibitors with or without cytotoxic T-lymphocyte-associated protein 4 (CTLA-4) blockade. Consistent with this concept, the randomized phase 3 ASCENT-04/KEYNOTE-D19 trial in previously untreated PD-L1-positive advanced triple-negative breast cancer demonstrated that replacing standard chemotherapy with the TROP2-directed ADC sacituzumab govitecan (SG) while maintaining pembrolizumab improved outcomes: progression-free survival was longer with SG plus pembrolizumab (median 11.2 vs. 7.8 months; hazard ratio 0.65, 95% confidence interval 0.51–0.84; *p* < 0.001), accompanied by higher objective response rates (60% vs. 53%), higher complete response rates (13% vs. 8%), and a longer duration of response (median 16.5 vs. 9.2 months) [85].

The appeal of ADCs as complementary modalities also stems from a key mechanistic feature beyond targeted cytotoxicity—immunostimulation, as shown in Figure 2. Extensive studies have suggested that several ADC payload classes can induce immunogenic cell death (ICD), in which dying tumor cells release or expose DAMPs—including surface calreticulin, extracellular adenosine triphosphate (ATP), and high-mobility group box 1 (HMGB1). These signals promote dendritic-cell activation and antigen cross-presentation, thereby enhancing tumor-specific T-cell priming and providing a mechanistic link between ADC-mediated cytotoxicity and antitumor immune responses [80]. For example, Bauzon et al. demonstrated that maytansine-bearing ADCs can elicit in vitro hallmarks of ICD—including cell-surface calreticulin exposure and the extracellular release of extracellular ATP and HMGB1—linking ADC-mediated cytotoxicity to immunostimulatory danger signaling. These ICD-associated signals were reported to occur selectively in antigen-positive target cells, supporting the concept that antibody-guided payload delivery can spatially restrict immunogenic cell death features to tumor cells, rather than normal tissues [81]. In another study of trastuzumab deruxtecan (T-DXd), accumulating preclinical and translational evidence indicates that the membrane-permeable DXd topoisomerase I payload can promote ICD and innate immune activation in both tumor models and ex vivo human tumor slice systems [82,83]. Consistent with this mechanism, datopotamab deruxtecan and its released DXd warhead have been reported to induce canonical ICD features—cell-surface calreticulin exposure, extracellular ATP, and HMGB1 release—supporting the broader concept that topoisomerase I-based ADC payloads can function as “chemo-immunotherapeutic” effectors, rather than purely cytotoxic agents [84].

Although these data were generated in breast cancer, they provide a clinically grounded proof-of-concept framework for exploring ADC–checkpoint inhibitor combinations in other tumor types, including cervical cancer. Conceptually, and as a hypothesis-generating model rather than a near-term clinical expectation, ADCs may be deployed as immune primers, with early cycles inducing damage-associated molecular pattern (DAMP) signaling and enhanced antigen presentation (and potentially adaptive PD-L1 upregulation), followed by PD-1/PD-L1 blockade to amplify and sustain tumor-reactive T-cell responses.

However, this proposition is indeed not a perfect model at all. Despite the biologic rationale supporting ADC–checkpoint inhibitor combinations, several factors may limit or negate synergistic benefit. Overlapping toxicities—particularly hematologic, dermatologic, or pulmonary events—may constrain the dose intensity or treatment duration when these agents are combined [86]. In addition, certain cytotoxic payloads may exert immunosuppressive effects at higher exposure levels, potentially attenuating rather than amplifying antitumor immune responses [87]. Tumor antigen heterogeneity and dynamic antigen loss may further limit ADC efficacy, reducing the magnitude of tumor cell death required to meaningfully enhance antigen presentation [88]. These considerations underscore that ADC–IO synergy remains biologically plausible but not assured, and careful clinical evaluation is required to determine optimal sequencing, dosing, and patient selection. As a result, the extent to which this sequential strategy translates into consistent clinical benefit in cervical cancer remains to be determined.

## 7. Limitations

### 7.1. Limitations of the Clinical Trials

While KEYNOTE-A18, -826, -158, -D19 and innovaTV-301 collectively support meaningful progress in cervical cancer therapy, several trial-design and implementation considerations temper interpretation and highlight gaps for translation. First, eligibility criteria and required performance status can enrich for fitter patients and may under-represent populations that are commonly encountered in practice (e.g., significant comorbidity burden, limited access to longitudinal follow-up, or prior treatment complexity), raising the possibility of selection effects that inflate apparent tolerability or adherence. Second, biomarker-driven inference remains constrained by heterogeneity: PD-L1 assessment varies by assay, scoring system, sampling site, and intratumoral variability, and a single baseline measurement may not capture dynamic evolution under therapy—limitations that are particularly relevant when interpreting outcomes in PD-L1–low/negative subgroups, where benefit may be attenuated or uncertain. Third, cross-trial comparisons (e.g., between concurrent CRT–IO strategies and systemic therapy trials in recurrent/metastatic disease) should be approached cautiously, given differences in disease setting, endpoints, prior therapy exposure, imaging schedules, and post-progression treatments, all of which can affect hazard ratios independently of intrinsic drug activity. Fourth, toxicity trade-offs are clinically consequential: adding immunotherapy to CRT may increase immune-related adverse events and treatment interruptions, whereas ADCs introduce distinct class toxicities (e.g., ocular and bleeding events for tissue factor-directed platforms) that can meaningfully impact quality of life, supportive-care burden, and real-world feasibility. Finally, hazard ratios alone do not fully capture unmet needs, including identifying patients with durable benefit versus early progression, defining optimal sequencing after IO or ADC exposure, developing strategies for PD-L1-low tumors and immunologically excluded TIME phenotypes, and ensuring equitable implementation in resource-limited settings. Collectively, these limitations underscore the need for prospective biomarker standardization, pragmatic/real-world studies, and mechanism-informed combination or sequencing trials that prioritize not only efficacy but also tolerability, access, and survivorship outcomes.

### 7.2. Toxicity, Fertility, and Survivorship Considerations Within the Conceptual Triad (CRT ± IO ± ADC)

Cervical cancer disproportionately affects patients of childbearing age, making survivorship, fertility, and quality-of-life outcomes central to treatment planning. In curative-intent settings, chemoradiation is associated with both acute toxicities (e.g., gastrointestinal and genitourinary symptoms, hematologic suppression) and late effects that can meaningfully affect long-term function, including bowel and bladder dysfunction, vaginal stenosis and sexual health sequelae, pelvic insufficiency fractures, and premature ovarian failure, depending on ovarian exposure. These toxicities underscore the importance of survivorship counseling and proactive supportive care, particularly as treatment intensification strategies evolve.

Immune checkpoint inhibitors introduce a distinct toxicity profile. Although many immune-related adverse events are manageable, a subset can be severe or persistent, and some—such as endocrinopathies—may require long-term hormone replacement and ongoing monitoring. From a survivorship perspective, these chronic toxicities and the need for longitudinal follow-up are especially relevant in younger populations. Likewise, antibody–drug conjugates can produce target- and payload-dependent adverse events that may affect feasibility and quality of life (e.g., ocular, neuropathic, dermatologic, hematologic, or bleeding risks, depending on the platform), often necessitating monitoring protocols and supportive-care resources.

Importantly, the cumulative toxicity profile of layering CRT, IO, and ADCs remains incompletely defined, and should be considered hypothesis-generating, rather than established. Overlap across modalities could plausibly increase treatment interruptions, prolong recovery, or amplify late effects, reinforcing the need for careful sequencing and safety-focused trial design. Accordingly, future studies evaluating multimodality regimens should incorporate prespecified acute and late toxicity monitoring, patient-reported outcomes, sexual health measures, fertility and pregnancy-related endpoints, and survivorship outcomes. Prospective registries may also be valuable to capture real-world long-term outcomes that may not be fully characterized in traditional efficacy-focused trials.

### 7.3. Global Disparities in Brachytherapy Access and Implementation

Beyond clinical trial and scientific limitations, cervical cancer care also faces global implementation barriers—most notably, limited access to brachytherapy—that substantially influence real-world outcomes. Although brachytherapy is a critical component of definitive treatment for locally advanced disease, many low- and middle-income settings face persistent barriers, including limited availability of after loaders and applicators, shortages of trained radiation oncologists/physicists and anesthesia support, restricted access to cross-sectional imaging for image-guided planning, and challenges in maintenance, quality assurance, and supply chains [89]. These constraints can lead to treatment delays, reduced dose delivery, or substitution with external-beam boosts, which may compromise local control and increase toxicity. Accordingly, global oncology efforts should prioritize scalable solutions—training and mentorship models, regional referral networks, equipment support and maintenance infrastructure, and pragmatic protocols—to expand safe brachytherapy delivery and ensure that advances in systemic therapy are implemented on a foundation of equitable access to definitive local treatment.

## 8. Conclusions

This review has outlined the goal of advancing all three modalities (chemotherapy, immunotherapy, and antibody–drug conjugates) toward a future in which each can be deployed as a first-line option and, when appropriate, as an effective monotherapy for a clearly defined patient population. For example, in patients strongly considering future pregnancy and with a desire to minimize exposure to broadly cytotoxic agents, IO may be preferred when tumor–immune features suggest a reasonable likelihood of benefit. Conversely, when patients do not meet established criteria suggesting immune sensitivity, ADCs may serve as a rational strategy to prime antitumor immunity before IO application, potentially converting immunologically “cold” tumors into more responsive states. Importantly, if systemic cytotoxicity remains a major concern, the targeted delivery paradigm of ADCs offers a path toward more selective tumor killing. As the field matures, innovations such as masked antibodies and dual-targeted designs may further reduce undesired off-tumor effects, minimizing cytotoxic exposure to the lowest achievable level—if not, in select contexts, this approaches functional elimination of traditional cytotoxicity.

While each modality should be sufficiently well established to “stand on its own” in the right clinical context, cervical cancer treatment is increasingly defined by how these approaches complement one another—balancing toxicity and quality of life. Chemotherapy remains a cornerstone because of its reliable, immediate tumor-reductive activity and its continued role as a backbone for combination regimens. IO has established a durable-benefit paradigm for a subset of patients, reinforcing the importance of tumor–immune context (e.g., HPV-driven antigenicity, immune infiltration, and checkpoint dependence) in shaping long-term outcomes. ADCs further expand the therapeutic landscape by delivering highly potent payloads in a targeted manner, offering a compelling strategy for patients with disease that has progressed beyond conventional approaches and, increasingly, a rational partner for IO through immunogenic cell stress and antigen release.

Across modalities, the central challenge is not a lack of active agents but the need for precision in patient selection, sequencing, and resistance management. Current practice continues to rely heavily on clinical factors and a limited biomarker toolkit; however, the heterogeneity of HPV-associated tumors, prior treatment exposure, and immune escape mechanisms suggests that “one-size-fits-all” escalation is unlikely to optimize benefit–risk for most patients. This heterogeneity also encompasses the clinically important subset of HPV-independent cervical cancers, which may follow distinct biology and may not fully benefit from paradigms that rely on viral antigenicity, underscoring the need for subgroup-specific biomarkers and trials. A forward-looking framework is to treat chemotherapy and ADCs as tumor debulking and immune-priming platforms, and IO as the durability engine—then evaluate how timing, dosing, and patient selection can convert short-lived responses into sustained disease control.

Future directions should prioritize: (i) biomarker-driven treatment allocation beyond single-marker approaches (integrating HPV/antigen features, immune phenotype, and dynamic in-treatment changes), (ii) rational sequencing and combination trials that predefine mechanism-based endpoints, (iii) resistance-focused studies with paired tissue and blood sampling, and (iv) toxicity-aware regimen design that preserves function and survivorship. Finally, because cervical cancer mortality is concentrated in low-resource settings, the real-world impact of these advances will depend on implementation—scalable diagnostics, practical treatment algorithms, and toxicity-monitoring capacity—alongside efforts to address the substantial costs and access barriers associated with IO and ADCs. Taken together, chemotherapy, IO, and ADCs form a converging therapeutic continuum—one that can be optimized through mechanism-guided sequencing and biomarker refinement to deliver deeper, longer-lasting control for patients with cervical cancer.

## Figures and Tables

**Table 1:** Cervical cancer biomarkers by clinical readiness.

Group	Clinically Actionable	Emerging	Exploratory/Research
Immune (IO-related)	PD-L1 (CPS)	TILs/CD8 density	IFN-γ/T-cell-inflamed signatures; spatial profiling; TCR clonality
Genomic instability	dMMR/MSI-H	TMB-high	APOBEC signatures; aneuploidy/CIN metrics
Targets (ADC/targeted)	—	Tissue factor (TF); HER2; TROP2	Target heterogeneity (H-score/%+); antigen loss
Clinical surrogates	FIGO stage; nodes; tumor size	Early imaging response; circulating HPV DNA	Radiomics; composite risk models

**Table 2:** CRT optimization: high-level principles relevant to combination strategies.

Domain	High-Level Principle	Why It Matters for CRT ± IO/ADC
Staging/selection	Use modern imaging to define extent (local + nodes)	Aligns patients to appropriate backbone and trial eligibility
Radiation delivery	Use conformal techniques and image guidance when available	Reduces toxicity → improves completion of multimodality regimens
Brachytherapy	Ensure timely, high-quality brachytherapy completion	Major determinant of local control; delays undermine outcomes
Overall treatment time	Avoid unplanned breaks; streamline logistics	Prolongation can reduce tumor control; impacts sequencing of systemic agents
Concurrent chemo	Use radiosensitizing chemotherapy as standard backbone when feasible	Sets the baseline against which added systemic agents are evaluated
Toxicity mitigation	Proactive supportive care (GI/GU, hematologic, renal)	Preserves dose intensity and feasibility of adding IO/ADC
Quality metrics	Track completion, dose, and timing	Enables comparability across studies and real-world implementation

**Table 3:** Stage-anchored framework for modality selection and sequencing.

FIGO-Based Setting	Backbone Modality	PD-L1 (CPS)	TIME Phenotype	HPV Biology	ADC Targets	Practical Takeaway
Early stage (localized)	Surgery ± adjuvant therapy	Optional	Mostly investigational	Contextual	Trial-based	Biomarkers mainly support risk stratification and trial eligibility; systemic sequencing usually not biomarker-driven
Locally advanced	Definitive CRT	±	Investigational	Contextual	Trial-based	CRT remains the anchor; biomarkers guide trial selection and support hypothesis-generating intensification (CRT ± IO and/or ADC)
Recurrent/metastatic	Systemic therapy	Decision-guiding	Investigational	Contextual	Emerging	PD-L1 most directly informs IO use where applicable; ADC targets inform ADC selection/clinical trials; sequencing limited by efficacy/toxicity data

## Data Availability

No new data were created or analyzed in this study. Data sharing is not applicable to this article.

## Abbreviations

The following abbreviations are used in this manuscript:

ADC	Antibody–drug conjugate
ATP	Adenosine triphosphate
CIN	Cervical intraepithelial neoplasia
CCRT	Concurrent chemoradiation
CPS	Combined positive score
CRT	Chemoradiation
CT	Computed tomography
CTLA-4	Cytotoxic T-lymphocyte-associated protein 4
DAMPs	Damage-associated molecular patterns
DC	Dendritic cell
DSS	Disease-specific survival
EBRT	External beam radiotherapy
FDG	Fluorodeoxyglucose
FIGO	Fédération Internationale de Gynécologie et d’Obstétrique
FRα	Folate receptor alpha
GZMB	Granzyme B
HMGB1	High-mobility group box 1
HPV	Human papillomavirus
HR	Hazard ratio
ICD	Immunogenic cell death
ICI	Immune checkpoint inhibitor
IFNG	Interferon gamma
IgG1κ	Immunoglobulin G1 kappa
IHC	Immunohistochemistry
IO	Immunotherapy
LAG-3	Lymphocyte activation gene 3
mc–Val–Cit–PABC	maleimidocaproyl–valine–citrulline–p-aminobenzyl carbamate
MMAE	Monomethyl auristatin E
MRI	Magnetic resonance imaging
ORR	Objective response rate
OS	Overall survival
PD-1	Programmed Death-1
PD-L1	Programmed Death-Ligand 1
PET	Positron emission tomography
PFI	Progression-free interval
PFS	Progression-free survival
PRF1	Perforin 1
RR	Relative risk
RT	Radiotherapy
SG	Sacituzumab govitecan
TCGA	The Cancer Genome Atlas
T-DXd	Trastuzumab deruxtecan
TF	Tissue factor
TIGIT	T-cell immunoreceptor with immunoglobulin and ITIM domains
TIL	Tumor-infiltrating lymphocyte
TIM-3	T-cell immunoglobulin and mucin-domain containing protein 3
TIME	Tumor-immune microenvironment
TROP2	Trophoblast cell-surface antigen 2
TV	Tisotumab vedotin
